# Reply to Comment on Choi, Y.-J., et al. Cellular Phone Use and Risk of Tumors: Systematic Review and Meta-Analysis. *Int. J. Environ. Res. Public Health* 2020, *17*, 8079

**DOI:** 10.3390/ijerph18063326

**Published:** 2021-03-23

**Authors:** Seung-Kwon Myung, Joel M. Moskowitz, Yoon-Jung Choi, Yun-Chul Hong

**Affiliations:** 1 Department of Cancer Biomedical Science, National Cancer Center Graduate School of Cancer Science and Policy, Goyang 10408, Korea; 2 Department of Family Medicine and Center for Cancer Prevention and Detection, Hospital, National Cancer Center, Goyang 10408, Korea; 3 Division of Cancer Epidemiology and Management, National Cancer Center Research Institute, Goyang 10408, Korea; 4 School of Public Health, University of California, Berkeley, Berkeley, CA 94720, USA; 5 Department of Preventive Medicine, College of Medicine, Seoul National University, Seoul 110-744, Korea; hierica8@snu.ac.kr (Y.-J.C.); ychong1@snu.ac.kr (Y.-C.H.); 6 Environmental Health Center, College of Medicine, Seoul National University, Seoul 03080, Korea; 7 Institute of Environmental Medicine, Seoul National University Medical Research Center, Seoul 03080, Korea

We appreciate Frank de Vocht and Martin Röösli’s interest [1] in our study and their recognition of the importance of conducting regular, systematic syntheses of the epidemiological evidence regarding the tumor risk from mobile phone (i.e., cellphone) use. They claim we made “peculiar decisions” in our review and meta-analysis of 46 case-control studies [2], and they tried to downplay the tumor risk from cellphone use by arguing that studies conducted by the Hardell group overestimated the risk compared to other studies. Finally, they criticized our assessment of study quality and funding source. In our response below we will demonstrate the weakness of their arguments.

First, de Vocht and Röösli disagreed with our decision to combine different types of tumors in our main meta-analysis. They argued that “it is not common practice to combine different outcomes with different aetiologies in one meta-analytic summary” citing Borenstein et al.’s textbook entitled “Introduction to Meta-analysis” [3]. However, when we thoroughly read the reference (pp. 357–364) that they cited, we could not find the same or similar description. Instead, in the introduction part (p. 357), it reads that “in the early days of meta-analysis (at least in its current incarnation). Robert Rosenthal was asked if it made sense to perform a meta-analysis, given that the studies differed in various ways, and whether the analysis amounted to combining apples and oranges. Rosenthal answered that combining apples and oranges makes sense if your goal is to produce a fruit salad.” If we expand this concept to our analysis, combining different types of tumors into just tumors might be possible. For example, it is known that smoking causes about 20% of all types of cancer and about 30% of all cancer deaths in the United States according to the American Cancer Society. These are the results from combining data from each organ-specific cancer. That is, even though each organ-specific cancer is a completely different outcome in terms of types of cancers, it is possible to combine them into the broader category, *cancer*. Furthermore, we did not combine different outcomes with “different aetiologies” in one meta-analytic summary, but combined different outcomes (in terms of each organ-specific tumor, but the same outcome in terms of tumor) in terms of “the same etiology”, *cellular phone use*. Moreover, we already presented findings from subgroup meta-analyses by type of cancer in Table S3 and summarized in the Results section, under the subtitle of 3.6. Use of Cellular Phones and Risk of Tumors in Subgroup Meta-analysis by Type of Tumor as follows: ‘Table S3 *shows the findings from the subgroup meta-analyses by type of tumor. There was no statistically significant association between cellular phone use and tumor risk in most subgroup meta-analyses. Increased tumor risk was found for malignant brain tumors only in the Hardell studies (OR, 1.35; 95% CI, 1.06 to 1.73; n = 5; I2 = 53.9%).’* There are other many examples that employ this approach. The association between smoking and cardiovascular disease is another typical example. Cardiovascular diseases include both coronary artery disease such as angina pectoris and myocardial infarction and cerebrovascular disease, which are apparently different outcomes in terms of types of organs (heart and brain) affected. Additionally, since our current study is an update of our widely-cited meta-analysis published in the *Journal of Clinical Oncology* in 2009 [4], we followed the protocols employed in our earlier peer-reviewed paper.

Second, de Vocht and Röösli claimed that we failed to notice “that both the INTERPHONE-related studies and miscellaneous studies are largely in agreement and do not point to an excess cancer risk from mobile phone use. Evidence of large excess cancer risks are almost exclusively based on the studies by the Hardell group...” [1]. We acknowledge that the current meta-analysis did not perform detailed subgroup meta-analyses by lifetime cellular phone use for each type of tumor. However, evidence of large excess risks is also shown in the INTERPHONE and miscellaneous case-control studies. The previous 13-nation INTERPHONE study reported a significantly elevated risk of glioma for 1640 or more hours of lifetime mobile phone use (OR = 1.40, 95% CI 1.03–1.89) [5]. Moreover, several follow-up papers using INTERPHONE data found even stronger evidence of an association between heavy mobile phone use and glioma risk [6,7,8]. In addition to the Hardell group [9,10,11,12], several INTERPHONE and miscellaneous case-control studies found significant associations between heavy or long-term mobile phone use and increased risk of acoustic neuroma [13,14,15,16]. In the meantime, it is likely that all the INTERPHONE-related case-control studies underestimated the tumor risk from heavy cellphone use because they have 11 serious design flaws; eight of which bias the results toward the null [17]. For example, all the INTERPHONE-related studies relied on self-reports of cellphone use which have been shown to have large random recall errors that bias risk estimates toward the null [18]. Moreover, some studies have been plagued with poor participation rates and some with differential participation rates. For example, the 13-nation INTERPHONE study had low participation rates, especially among controls which introduced a selection bias that lowered risk estimates [17]. To adjust for this bias, the investigators published the results of secondary analyses in Appendix 2 of their paper [5]. In 2013, the Hardell group [11] published a study that controlled for factors to make their glioma analysis comparable to the INTERPHONE study [5] by using the same age range and by excluding use of cordless phones. They obtained a similar result for heavy (1640 h or more) lifetime cellphone use (OR = 1.75, 95% CI: 1.02–3.00) as INTERPHONE found in Appendix 2 (OR = 1.82, 95% CI: 1.15–2.89) [5]. Moreover, a “miscellaneous study”, the French CERENAT study, found an even stronger association with glioma risk with fewer cumulative hours of cellphone use (≥896 h; OR = 2.89, 95% CI = 1.41–5.93) than either Hardell or Interphone [18].

Third, they claimed that “relative excess risks of 90% (30–170%) and 70% (4–180%) reported by the Hardell group (Table 1 and Figure 2) associated with any mobile phone use are implausible (sic) high, and do not triangulate with evidence from other epidemiological sources, such as prospective cohort studies and incidence trends.” The high relative excess risks reported by the Hardell group is due to the inclusion of users exposed to more cellphone radiation. Measures of cellphone use (e.g., number of cumulative hours of call time) serve as proxies for exposure to cellphone radiation. A recent study found that a cellphone’s emissions can vary by four orders of magnitude depending upon the signal strength from the nearest cell tower [19]. Thus, two people with the same amount of cellphone use and the same cellphone model can experience very different cumulative exposure to cellphone radiation depending upon their proximity to cellphone towers. The Hardell group reported that “adaptive power control regulates the output power level from cellular telephones, mainly the digital system, with the highest level in areas with a long distance between base stations.” They found greater brain tumor risk for those who used digital cellphones >5 years in rural areas (OR = 3.2, 95% CI 1.2–8.4) than in urban areas (OR = 0.9, 95% CI 0.6–1.4) [20]. Thus, the Hardell group’s greater cellphone-tumor risk estimates could be explained by the inclusion of more rural users in their studies. To support their argument that mobile phone use does not increase tumor risk, de Vocht and Röösli cited two prospective studies, the Danish Cohort Study [21] and the Million Women Study [22]. Unfortunately, neither study was originally designed to assess the effects of mobile phone use; hence, both had poor measures of cellphone use. Plus, the Danish study classified as non-cellphone users many heavy cellphone users who had cellphones purchased by their businesses which reduced risk estimates below 1 [23]. Although the Million Women Study did not find an association between cellphone use and glioma risk, the study found that those who used cellphones ≥10 years (RR = 2.46, 95% CI = 1.07–5.64) had a greater risk of acoustic neuroma, a nonmalignant tumor, and among cellphone users, the risk increased with duration of use (*p* = 0.03) [22]. We already mentioned these limitations of the two prospective studies in our Discussion Section. Moreover, de Vocht and Röösli cited an Australian study which found no increase in brain tumor incidence in years of substantial cellphone use [24]. However, the incidence of certain brain tumor subtypes, e.g., glioblastoma or glioma in the frontal and temporal lobes, have increased during recent years in other countries including the U.S., Sweden, and the United Kingdom [25,26,27,28].

Fourth, we disagree with de Vocht and Röösli that the “Hardell studies should have been classified as being of comparable quality to the other case-control studies in this review, at most”. After reviewing these studies and the INTERPHONE studies conducted in Sweden, we believe that the “serious concerns” raised by de Vocht and Röösli about the Hardell studies are not supported by the evidence. The differences in response rates seem attributable to the higher quality methods employed by the Hardell study group [14,29,30].

Finally, they claimed that “Only the Hardell studies received direct funding from interest groups such as the telecom industry and pressure groups, but this was not reported by Choi et al”. We are sorry that we did not recognize that. However, although the Hardell studies received funding from the telecom industry and might be associated with the interests of pressure groups, their conclusions do not correspond with the interest of the telecom industry.

Although our updated meta-analysis of case-control studies suggested significant evidence linking cellular phone use to increased tumor risk, we are unable to draw a definitive conclusion on this topic because case-control studies potentially suffer from recall bias and selection bias. Although it is not feasible to conduct randomized controlled trials (RCTs) with humans on this topic, two recent RCTs found increased tumor risk from long-term exposure to cellphone radiation in an animal model providing evidence of a causal effect [31,32]. Moreover, the same types of cells (glial, Schwann) were affected in these two studies as in the case-control studies.

Unlike the INTERPHONE and miscellaneous studies, the Hardell studies achieved high participation rates and smaller differences in response rates between the cases and controls. In contrast, the INTERPHONE-related studies have serious shortcomings and limitations in that they were partly funded by the mobile industry, had poor methodological quality, showed larger differences in response rates between the case and control groups, and did not use blinding at interview. We appreciate de Vocht and Röösli’s comments on our study and think that further prospective cohort studies using exact data on the time spent on cellular phones along with actual measures of the cellphone’s electromagnetic field emissions are warranted to confirm our findings.
